# The Major Histocompatibility Complex in Transplantation

**DOI:** 10.1155/2012/842141

**Published:** 2012-06-20

**Authors:** Marco Antonio Ayala García, Beatriz González Yebra, Andrea Liliana López Flores, Eduardo Guaní Guerra

**Affiliations:** ^1^Investigación, Hospital Regional de Alta Especialidad del Bajío, Blvd. Milenio No. 130, San Carlos/La Roncha, 37660 León, GTO, Mexico; ^2^HGSZ No.10 del Instituto Mexicano del Seguro Social Delegación Guanajuato, Cantador No. 17, 36000, GTO, Mexico; ^3^Departamento de Medicina y Nutrición,Universidad de Guanajuato, 20 de Enero No. 929, 37000 León, GTO, Mexico

## Abstract

The transplant of organs is one of the greatest therapeutic achievements of the twentieth century. In organ transplantation, the adaptive immunity is considered the main response exerted to the transplanted tissue, since the principal target of the immune response is the MHC (major histocompatibility complex) molecules expressed on the surface of donor cells. However, we should not forget that the innate and adaptive immunities are closely interrelated and should be viewed as complementary and cooperating. When a human transplant is performed, HLA (human leukocyte antigens) molecules from a donor are recognized by the recipient's immune system triggering an alloimmune response Matching of donor and recipient for MHC antigens has been shown to have a significant positive effect on graft acceptance. This paper will present MHC, the innate and adaptive immunities, and clinical HLA testing.

## 1. Introduction

The primary function of the immune system is to protect the host from infectious microbes in its environment. This system has evolved over millions of years, in response of coexistence with microorganisms. Basically, the system can be divided in two components, the innate and adaptive immunities.

## 2. Innate and Adaptive Immunities

The innate also called natural immunity refers to a nonspecific response that involves the recruitment of diverse components of the immune system such as macrophages, neutrophils, natural killer cells (NK cells), cytokines, several cellular receptors, complement components, cytokines, Toll-like receptors (TLRs), and antimicrobial peptides (AMPs). This response is phylogenetically older in comparison to the adaptive immunity, which involves recognition of specific antigen, conferring both specificity and a memory effect [1]. The main effectors of the adaptive immunity are the T and B cells. T cells recognize antigen in the form of peptide bound to major histocompatibility complex (MHC) molecules [2]. B cells have immunoglobulin receptors that recognize the antigenic portions of determined molecules [3].

In organ transplantation, the adaptive immunity is considered the main response exerted to the transplanted tissue, since the principal target of the immune response is the MHC molecules expressed on the surface of donor cells. However, we should not forget that the innate and adaptive immunities are divided only by educational purposes, since both are codependent. For example, T-cell activation leads to the production of cytokines and chemokines which in turn may recruit components of the innate immunity like NK cells or macrophages [4]. Furthermore, local tissue production of complement components seems to be essential for full T-cell activation [4], and some AMPs like defensins and cathelicidin have chemoattractant properties on T lymphocytes [5].

## 3. Discrimination of Self from Nonself

Because the immune system uses many different effector mechanisms to destroy the broad range of microbial cells and particles that it encounters, it is critical for the immune response to avoid unleashing these destructive mechanisms against its own tissues. This avoidance of destruction of self-tissues is referred to as self-tolerance. Mechanisms to avoid reaction against self-antigens are expressed in many parts of both the innate and the adaptive immune responses. Failure of self-tolerance underlies the broad class of autoimmune diseases [1]. Unfortunately, transplanted tissues from individuals of the same species (allogenic) or different species (xenogeneic) are recognized as nonself, causing graft rejection. The process by which the immune system recognizes pathogens, tumors, and transplantation antigens involves the same antigen recognition molecules.

## 4. Transplantation Antigens

The rejection response to grafted tissue is caused by cell surface molecules that induce an antigenic stimulus. A wide variety of transplantation antigens have been described, including the MHC molecules, minor histocompatibility antigens, ABO blood group antigens, and monocytes/endothelial cell antigens. The minor histocompatibility antigens are processed peptides derived from cellular antigens that are presented by MHC molecules but are not derived from the MHC [6]. ABO compatibility is of much less importance than MHC compatibility in graft survival. However, ABO incompatibility can result in hyperacute rejection of primarily vascularized grafts, such as kidney and heart [7]. As we mentioned before, the principal target of the transplantation immune response is the MHC molecules expressed on the surface of donor cells.

## 5. The Major Histocompatibility Complex

**Figure 1:**
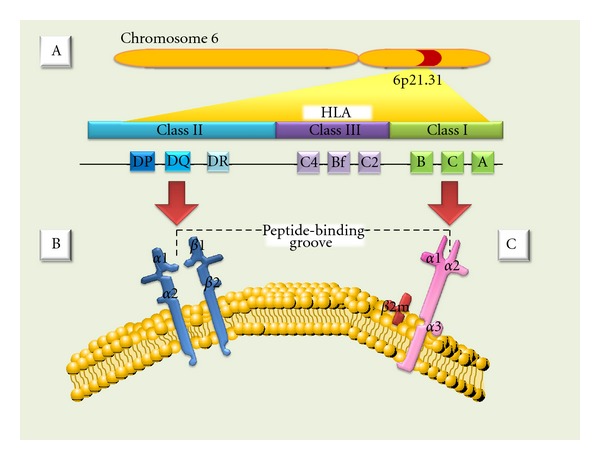
(A) MHC (major histocompatibility complex). (B) Class II antigens are expressed only on B lymphocytes, activated T lymphocytes, monocytes, macrophages, Langerhans cells, dendritic cells, endothelium, and epithelial cells. They are heterodimers composed of noncovalently associated *α* and *β* polypeptide chains chains encoded by genes of the HLA-D region. (C) Class I MHC antigens are present on all nucleated cells and are composed of a 45-kd transmembrane *α* heavy chain encoded by genes of the HLA-A, HLA-B, or HLA-C loci on chromosome 6.

According to their relative potencies in eliciting rejection, the major antigens in mammalian species are encoded by a closely linked series of genes called MHC. In humans, these genes reside in the short arm of chromosome 6 (Figure 1(A)). Organs transplanted between MHC identical individuals are readily accepted, whereas organs transplanted between MHC antigen-mismatched individuals are rejected in the absence of immunosuppressive therapy [8, 9]. Since the MHC was first defined in mice by Gorer and Snell [10, 11], the World Health Organization Nomenclature Committee has named HLA (human leukocyte antigen) to the human MHC [12].

The HLA complex genes and their protein products have been divided into three classes (I, II, and III) on the basis of their tissue distribution, structure, and function [13, 14]. MHC class I and II genes encode codominantly expressed HLA cell surface antigens, and class III genes encode several components of the complement system; all share important roles in immune function [12]. Class I MHC antigens are present on all nucleated cells and are composed of a 45-kd transmembrane *α* heavy chain encoded by genes of the HLA-A, HLA-B, or HLA-C loci on chromosome 6; the *α* heavy chains are associated noncovalently with a 12-kd protein, *β*2-microglobulin, encoded by a gene on chromosome 15 (Figure 1(C)) [13]. Additional (nonclassical) class I molecules, like those encoded by the HLA-E, -F, -G, -H loci, have been described and show limited variability and tissue distribution. The precise functions of these molecules are not yet clear, although they have been implied in presenting carbohydrate and peptide fragments to *γδ* T cells and mother's immunological tolerance of the fetus [14–17]. MHC class II antigens are expressed only on B lymphocytes, activated T lymphocytes, monocytes, macrophages, Langerhans cells, dendritic cells, endothelium, and epithelial cells [18]. Class II molecules are heterodimers composed of noncovalently associated *α* and *β* polypeptide chains chains encoded by genes of the HLA-D region (Figure 1(B)). There are 3 major class II proteins designated, HLA-DP, HLA-DQ, and HLA-DR. Class III genes are located between the HLA-B and HLA-D loci and determine the structure of three components of the complement system: C2, C4, and factor B [13, 19]. Class I MHC molecules present cytoplasm-derived peptides, or intracellular parasites, principally viruses; whereas MHC class II molecules bind peptides derived from extracellular proteins [1]. HLA class I and II molecules are recognized by CD8 and CD4 positive T cells, respectively [20–22]. Also, NK cells may recognize HLA classical and nonclassical type I molecules [23–25]. 

HLA antigens are inherited in a Mendelian dominant manner. HLA genes are almost always inherited together, thus the antigens of the entire HLA region inherited from one parent collectively are called haplotype. Because chromosome 6 is an autosome (a chromosome with two pairs), all individuals have two HLA haplotypes (one for each chromosome) [12]. According to this, any sibling pair has a 25% chance of inheriting the same two parental haplotypes, a 50% chance of sharing one haplotype, and a 25% chance of having two completely different haplotypes. All children are haploidentical with each parent [6].

Since the biologic function of the HLA molecules is presenting endogenous and exogenous antigens, they manifest high structural polymorphism. Until 2010, 2558 HLA class I and II alleles have been recognized (Table 1) [26]. Mutations in microbial antigens might permit the microbe to avoid binding (and, consequently, recognition) by a few HLA alleles, but no mutations will permit the microbe to avoid recognition broadly throughout the population; assuring then, the continuity of species in the presence of pandemic infection [12].

In transplantation immunology, the major impact in graft loss comes from the effects of HLA-B and -DR antigens [27]. There also appears to be a temporal HLA mismatching effect. HLA-DR mismatch effect is the most important in the first 6 months after transplantation, the HLA-B effect emerges in the first 2 years, and HLA-A mismatches have a deleterious effect on long-term graft survival [28–32].

## 6. The Allogeneic Immune Response

The phenomenon by which the recipient immune system reacts with donor antigens that are considered to be “non-self” is named allorecognition. The main and strongest responses to alloantigens are mediated by host T cells, which recognize peptide antigens presented in the context of MHC, by antigen-presenting cells (APCs). However, evidence that the innate alloimmunity has an important role in graft rejection has recently been proposed by Land and coworkers [33, 34]. They state in their “Injury Hypothesis” that initial allograft injury reflected by reactive oxygen species (ROS) during reperfusion is associated with generation of DAMPs (meaning damage-associated molecular patterns) such as heat shock proteins (HSP) and hyaluronan fragments (fHA) among others, all of which are recognized by TLR4 and/or TLR2. Subsequent TLR4- and TLR2-triggered signaling pathways utilize adaptor proteins including MyD88 (myeloid differentiation marker 88), which in turn initiate downstream signaling pathways that lead to activating the 3 master transcription factors NF-*κ*B (nuclear factor-kappa B), AP-1 (activator protein-1), and IRF-3 (interferon regulatory factor 3). NF-*κ*B seems mainly to be responsible for maturation of donor-derived and recipient-derived dendritic cells, which represents the bridge to development of an adaptive alloimmune response that results in rejection [35]. Certainly, further studies are needed to determine the extension and importance of this branch of the immune system in transplant rejection and/or tolerance.

**Figure 2:**
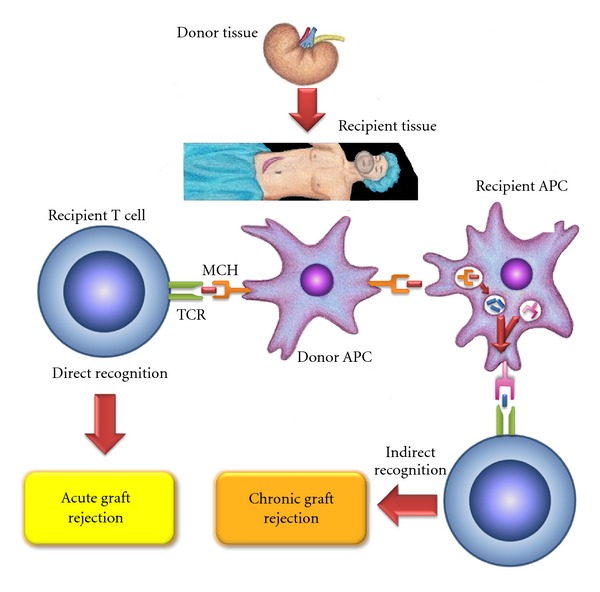
Allogeneic immune response: this could happen by three recognizing mechanisms: first, an indirect recognition: this type of mechanism has a dominant role in chronic rejection; second, a direct recognition: this mechanism determines a strong immune response in the acute rejection; third mechanism, a “semi-direct” recognition that could be mediated by immunoglobulin-like receptors of natural killer (NK) cells and can mediate potent acute rejection.

In adaptive allogenic immune response, the foreign or donor antigen presentation to T cells may occur by three ways (Figure 2) [36]: (1) indirect recognition: donor's HLA molecules can be processed by APC (antigen presenting cells) from a receptor, then they are fractionated into peptides as well as other bacterial antigens and are presented according to the same route as the HLA in the receptor. This type of mechanism has a dominant role in chronic rejection [37–41]; (2) direct recognition: the donor's HLA molecules can be recognized directly on the donor-presenting cells, without requiring antigen processing by receptor. In these circumstances, it could be said that the receptor identifies the foreign HLA molecule as an own molecule with a foreign peptide. This mechanism determines a strong immune response in the acute rejection [37, 38, 42–50]; (3) a third mechanism could be mediated by immunoglobulin-like receptors of natural killer (NK) cells. In this mechanism, the activation of NK receptors promotes the inactivation of NK cells and cytotoxic T lymphocytes as well. These receptors recognize polymorphic sequences of HLA-C, -B, or -A in the target cells. The absence of these sequences in the cell would make them sensitive to cytolysis and therefore the loss of tolerance [51–56].

Recently, it was shown that both naïve and memory CD4^+^ and CD8^+^ T cells are frequently cross-reactive against allogeneic HLA molecules and that this allorecognition exhibits exquisite peptide and HLA specificity. Such advances in the understanding of the immunogenetics of allorecognition have led some researches to suggest a new model for allorecognition whereby the majority of T cell alloresponses may occur via direct recognition (cross-reactivity) by thymically educated naïve and memory T cells against allogeneic HLA molecules presenting self-peptides. According to this model, thymically educated T cells are commonly and specifically allo-HLA reactive and are activated by viral infection or vaccination to become alloreactive memory T cells which are a major barrier to successful tolerance [57]. 

## 7. Clinical HLA Testing

To support the transplant programs, several clinical laboratories perform various HLA tests, including HLA typing of the recipient and the donor, screening of HLA antibodies in the recipient, and detection of antibodies in the recipient that are reactive with lymphocytes of a prospective donor (cross-matching).

Historically, HLA typing was conducted by serologic testing by using antiserum in complement-dependent cytotoxic assays. Recently, more precise DNA-based HLA typing methods using molecular techniques, such as sequence-specific oligonucleotide probe hybridization, sequence-specific primer amplification, sequencing-based typing, and reference strand-based conformation analysis, have been developed and are frequently used [58]. 

There is a clear relationship between the degree of HLA matching and kidney graft survival in transplants from living-related donors. Simultaneous analysis of 5,262 one haplotype-matched living-related allografts, and 973 HLA identical allografts showed 10-year projected survival rates of 52% and 73% and graft half-lives of 11.9 and 23.6 years, respectively. Conversely, the influence of HLA matching on the survival of liver and thoracic organs is yet uncertain [59].

To avoid hyperacute rejection, it is very important to identify recipient anti-HLA antibodies to antigens expressed on donor with blood cells. The pioneer method to detect such antibodies, the complement-dependent cytotoxicity (CDC), has been gradually replaced by more-sensitive solid-phase assays, such as the enzyme-linked immunosorbent assay and the bead-based technology (i.e., flow cytometry: FlowPRA and Flow Analyzer: Luminex). However, the new techniques have been associated with decreased specificity, and some non-HLA antigens with no clinical relevance have been able to give a positive crossmatch [60]. These “false-positive” antibody results have as a consequence a decreased chance of the patient to receive an organ by way of exchange organizations, thus decreasing chances for the patient [61]. Thus, the experts recommend that the information these tests provide should complement that of the direct CDC assay.

## 8. Conclusions

Development of the field of organ and tissue transplantation has accelerated remarkably since the human major histocompatibility complex (MHC) was discovered in 1967. However, has been elusive avoid the graft rejection. This is due to that the transplantation immunobiology is very complex, because of the involvement of several components such as antibodies, antigen presenting cells, helper and cytotoxic *T* cell subsets, immune cell, surface molecules, signaling mechanisms, and cytokines, which play a role in innate and adaptive immunities.

## Figures and Tables

**Table 1:** List of all recognized serological and cellular HLA specificities.

Locis	Class I				Class II		
	A	B		C	DR	DQ	DP
	A1	B5	B50 (21)	Cw1	DR1	DQ1	DPw1
	A2	B7	B51 (5)	Cw2	DR103	DQ2	DPw2
	A203	B703	B5102	Cw3	DR2	DQ3	DPw3
	A210	B8	B5103	Cw4	DR3	DQ4	DPw4
	A3	B12	B52 (5)	Cw5	DR4	DQ5 (1)	DPw5
	A9	B13	B53	Cw6	DR5	DQ6 (1)	DPw6
	A10	B14	B54 (22)	Cw7	DR6	DQ7 (3)	
	A11	B15	B55 (22)	Cw8	DR7	DQ8 (3)	
	A19	B16	B56 (22)	Cw9 (w3)	DR8	DQ9 (3)	
	A23 (9)	B17	B57 (17)	Cw10 (w3)	DR9		
	A24 (9)	B18	B58 (17)		DR10		
	A2403	B21	B59		DR11 (5)		
	A25 (10)	B22	B60 (40)		DR12 (5)		
	A26 (10)	B27	B61 (40)		DR13 (6)		
Alleles	A28	B2708	B62 (15)		DR14 (6)		
	A29 (19)	B35	B63 (15)		DR1403		
	A30 (19)	B37	B64 (14)		DR1404		
	A31 (19)	B38 (16)	B65 (14)		DR15 (2)		
	A32 (19)	B39 (16)	B67		DR16 (2)		
	A33 (19)	B3901	B70		DR17 (3)		
	A34 (10)	B3902	B71 (70)		DR18 (3)		
	A36	B40	B72 (70)		DR51		
	A43	B4005	B73		DR52		
	A66 (10)	B41	B75 (15)		DR53		
	A68 (28)	B42	B76 (15)				
	A69 (28)	B44 (12)	B77 (15)				
	A74 (19)	B45 (12)	B78				
	A80	B46	B81				
		B47	B82				
		B48	Bw4				
		B49 (21)	Bw6				
